# Circulating monocytes expressing senescence‐associated features are enriched in COVID‐19 patients with severe disease

**DOI:** 10.1111/acel.14011

**Published:** 2023-11-15

**Authors:** Y. Lin, D. F. Postma, L. S. Steeneken, L. S. Melo dos Santos, J. L. Kirkland, J. M. Espindola‐Netto, T. Tchkonia, M. Borghesan, H. R. Bouma, M. Demaria

**Affiliations:** ^1^ European Research Institute for the Biology of Ageing (ERIBA) University Medical Center Groningen (UMCG), University of Groningen (RUG) Groningen Netherlands; ^2^ Department of Internal Medicine and Infectious Diseases University Medical Center Groningen (UMCG) Groningen The Netherlands; ^3^ Clinical Pharmacy & Pharmacology University Medical Center Groningen (UMCG) Groningen The Netherlands; ^4^ Department of Physiology and Biomedical Engineering Mayo Clinic Rochester Minnesota USA; ^5^ Robert and Arlene Kogod Center on Aging Mayo Clinic Rochester Minnesota USA

**Keywords:** ageing, cellular senescence, COVID‐19, monocytes, SARS‐CoV‐2, SASP

## Abstract

Accurate biomarkers for predicting COVID‐19 severity have remained an unmet need due to an incomplete understanding of virus pathogenesis and heterogeneity among patients. Cellular senescence and its pro‐inflammatory phenotype are suggested to be a consequence of SARS‐CoV‐2 infection and potentially drive infection‐dependent pathological sequelae. Senescence‐associated markers in infected individuals have been identified primarily in the lower respiratory tract, while little is known about their presence in more easily accessible bio‐specimens. Here, we measured the abundance of senescence‐associated signatures in whole blood, plasma and peripheral blood mononuclear cells (PBMCs) of COVID‐19 patients and patients without an infection. Bulk transcriptomic and targeted proteomic assays revealed that the level of senescence‐associated markers, including the senescence‐associated secretory phenotype (SASP), is predictive of SARS‐CoV‐2 infection. Single‐cell RNA‐sequencing data demonstrated that a senescence signature is particularly enriched in monocytes of COVID‐19 patients, partially correlating with disease severity. Our findings suggest that monocytes are prematurely induced to senescence by SARS‐CoV‐2 infection, might contribute to exacerbating a SASP‐like inflammatory response and can serve as markers and predictors for COVID‐19 and its sequelae.

## Graphical abstract

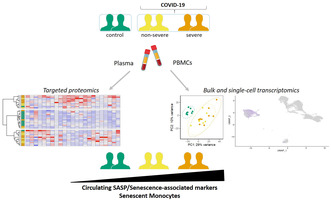

**Abbreviations**
AUC: area under the curve
cMono: classical monocytes
COMBAT: covidCOVID‐19 multi‐omics blood atlas
DEG: differentially expressed genes
GO: gene ontology
HV: healthy volunteer
ncMono: non‐classical monocytes
PBMCs: peripheral blood mononuclear cells
PC: principal component
SASP: senescence‐associated secretory phenotype
UMAP: uniform manifold approximation and projection

The outbreak of the COVID‐19 pandemic caused by the SARS‐CoV‐2 virus has led to a worldwide health crisis. The viral pathogenesis triggers a complex interplay between host immune responses and viral factors, leading to dysregulated immune activation and cellular damage. One of the significant consequences of SARS‐CoV‐2 infection is the alteration in the composition and function of various blood components (COvid‐19 Multi‐omics Blood ATlas (COMBAT) Consortium, 2022), associated with an ineffective yet overwhelming immune response to the infection, organ failure and poor clinical outcomes.

Various types of lung cells can undergo senescence in response to SARS‐CoV‐2, causing the release of inflammatory and tissue damage factors part of the senescence‐associated secretory phenotype (SASP) (Lee et al., 2021) which can result in long‐term tissue damage and fibrosis (Borghesan et al., 2020; Lee et al., 2021; Schmitt et al., 2023; Tsuji et al., 2022). The extent of white blood cells proliferation, activation and senescence among COVID‐19 patients remains poorly understood.

We performed transcriptome and proteome analysis of samples part of the Acutelines biobank (see M&M). We included 12 non‐infected (control) individuals, 10 patients with non‐severe symptoms and 14 patients with severe symptoms based on average SpO_2_ values of 98.5%, 96.5% and 90.0%., respectively. The median ages of the three groups were 66, 65 and 69 years, and the male: female ratio was 1.4, 4 and 2.5 (for details see Table S1).

Using a customized panel of SASP markers (Tchkonia et al., 2013), we identified 23 proteins in plasma that met the minimal abundance criteria, displaying an overall higher expression in infected patients after accounting for age (Figure 1a, Table S1). Bulk RNA sequencing on white blood cells showed that control samples formed a tightly clustered group, while patient samples exhibited a more scattered pattern (Figure 1b). Differential gene expression analysis identified 1258 up‐regulated genes and 844 down‐regulated genes when comparing severe to the other groups, and 929 genes up‐regulated and 327 down‐regulated genes when comparing non‐severe to the control group. A total of 946 genes were commonly differentially regulated in both severe and non‐severe cases compared to control groups, with 735 genes up‐regulated and 211 genes down‐regulated (Table S2). Most significant biological functions of genes differentially expressed in infected patient samples were complement activation, phagocytosis, B‐cell activation and viral defence (Figure 1c).

**FIGURE 1:**
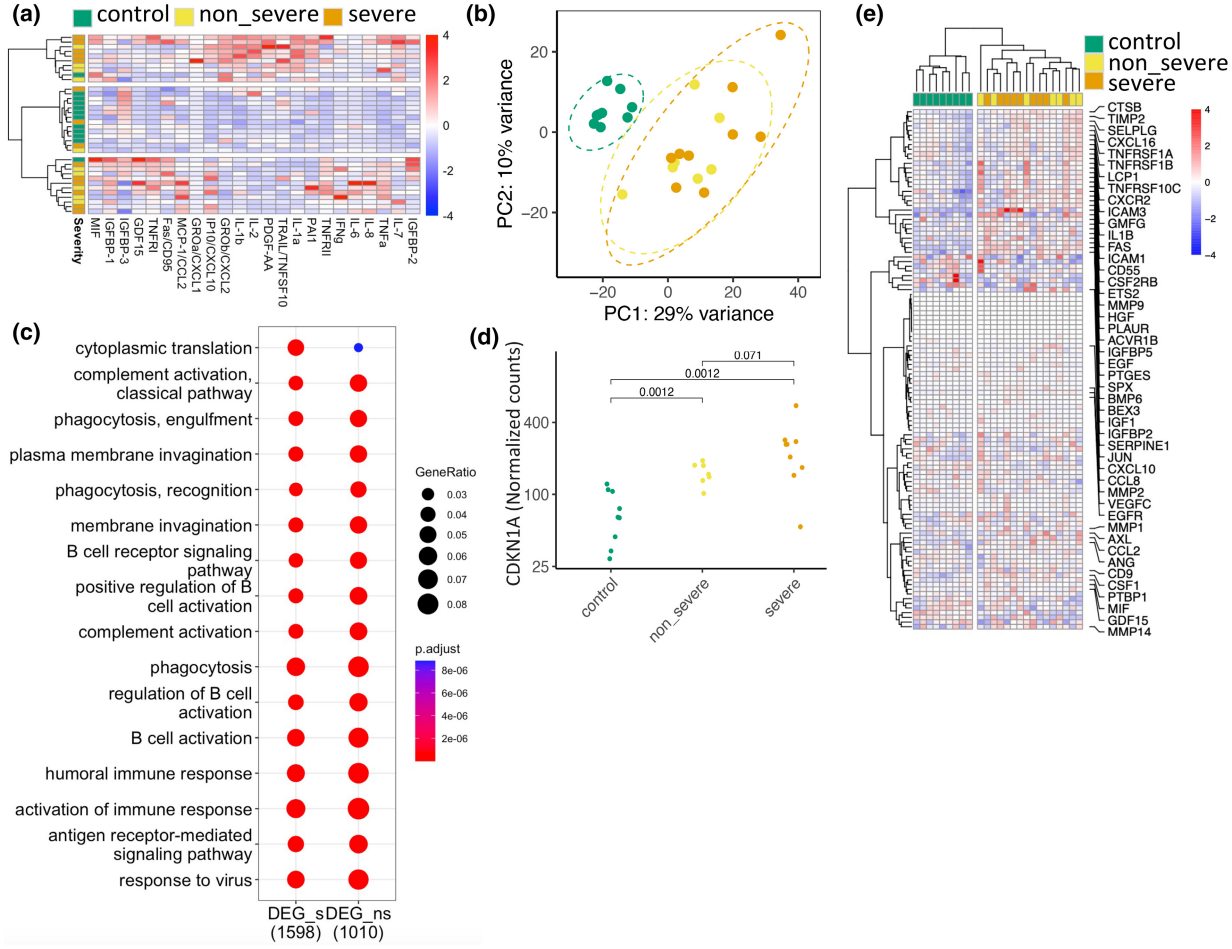
Whole blood transcriptome and plasma SASPs showed senescent signatures in COVID‐19 patients. (a) Heatmap of SASP proteins identified in plasma samples from healthy donors (green), patients with non‐severe symptoms (yellow) and patients with severe symptoms (tangerine). (b). Principal component analysis plot that shows the clustering of RNA‐Seq samples based on their gene expression levels. (c) Dot plot of the top 15 enriched GO terms based on the differentially expressed genes in non‐severe and severe patients when compared to control individuals. (d) Expression levels of the CDKN1A gene in relation to the severity of the disease. (e) Heatmap analysis of the SenMayo gene signature.

Expression of CDKN2A (p16) was not detected, but levels of CDKN1A (p21) correlated with disease severity (Figure 1d). A recently annotated gene signature of cell senescence, the SenMayo signature (Saul et al., 2022), was up‐regulated in the patient groups, but unable to discriminate between non‐severe and severe cases (Figure 1e).

In order to understand which cell type acquire senescent features, we used two publicly available single‐cell RNA‐Seq datasets (COMBAT Consortium, 2022; Wang et al., 2022). To assign cell identity, we employed an unsupervised clustering and reference‐based method with SingleR (Aran et al., 2019; Monaco et al., 2019), followed by manually checking the expression of known marker genes (Figure S1), resulting in the identification of 29 cell types (Figure 2a). As shown in the UMAP plot (Figure 2b), CDKN1A was specifically up‐regulated in the monocyte cluster. When comparing COVID‐19 patients to healthy donors, we found that cells from moderate and severe patients had relatively higher CDKN1A expression in classical monocytes, intermediate monocytes and non‐classical monocytes (Figure 2c) which correlated with an increased percentage of monocytes expressing G1‐phase‐associated gene signatures, but a lower percentage of S phase‐associated gene signatures with increasing disease severity (Figure 2d).

**FIGURE 2:**
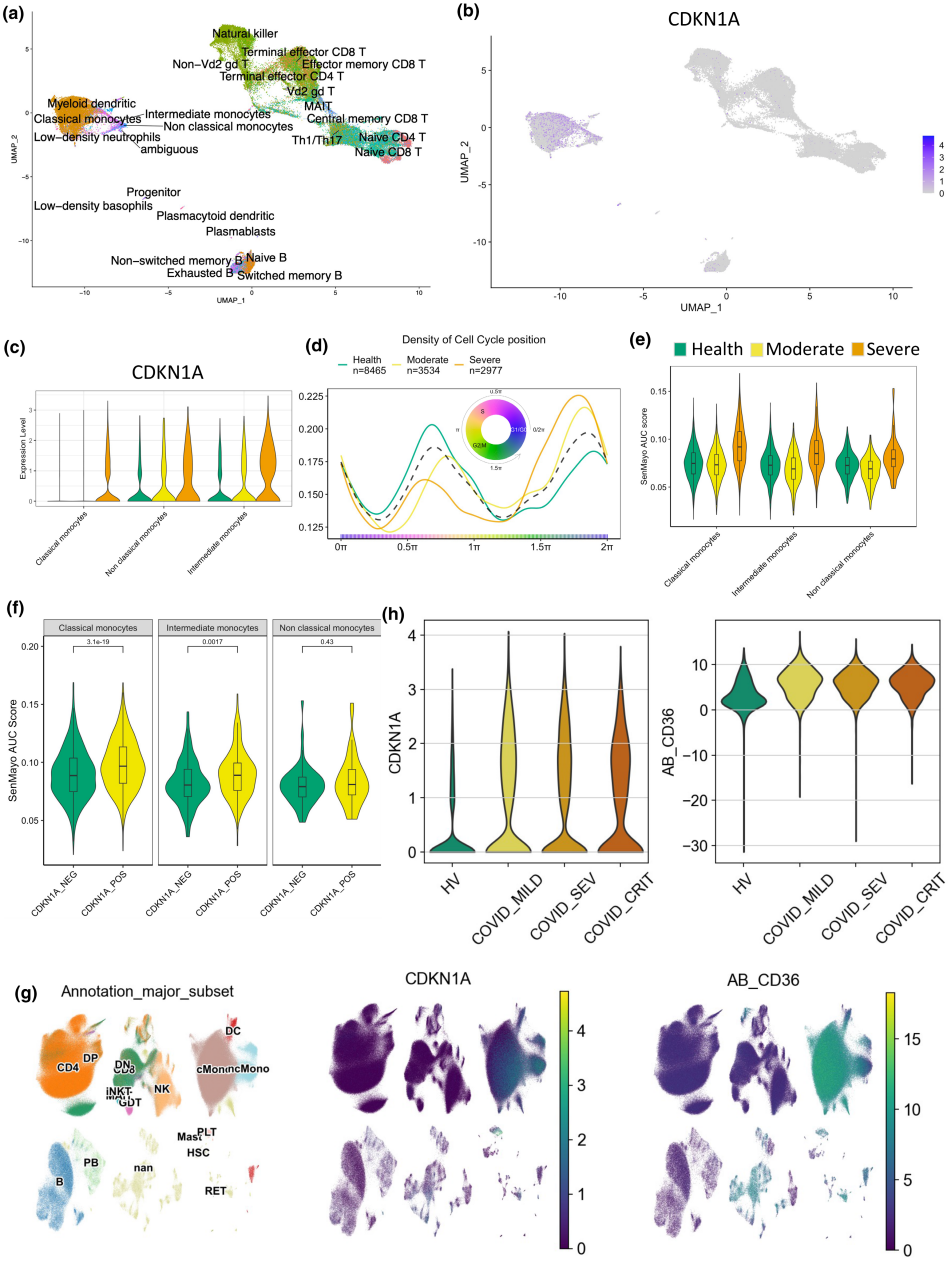
Identification of monocytes as the primary source of senescence in COVID‐19 patients, as evidenced by the up‐regulation of CDKN1A. (a) Uniform Manifold Approximation and Projection (UMAP) plot representing the identified cell types. (b) UMAP plot showing the expression of senescent marker gene CDKN1A in all cell types. The colour is labelled based on the expression level in log scale. (c) CDKN1A expression in monocytes. (d) Cell cycle position density plot for monocytes from samples with different disease severity. (E, F) Area under the curve (AUC) scores of the SenMayo gene list in monocytes (e), with a specific focus on the AUC scores of CDKN1A positive and negative monocyte cells (f). (g) UMAP plot of cell annotation results and expression levels of the senescent markers CDKN1A and CD36. The log‐normalized gene expression levels are represented by a colour scale. (h) Expression levels of CDKN1A and CD36 associated with disease severity. WHO severity categories include HV: Healthy Volunteer; COVID_MILD: COVID patient with mild symptoms; COVID_SEV: COVID patient with severe symptoms; COVID_CRIT: COVID patient with critical symptoms.

Expression of the SenMayo (Saul et al., 2022) signature was observed in monocytes from patients with severe disease (Figure 2e) (Aibar et al., 2017), and, using the gene set enrichment score AUCell, we observed a significant up‐regulation of the SenMayo signature expression in the CDKN1A‐positive cells (Figure 2f).

To evaluate whether a senescent profile could be observed on the surface of leukocytes of COVID‐19 patients, we used CD36, a newly emerged cell surface senescence marker (Moiseeva et al., 2023; Rossi & Abdelmohsen, 2021). Using a multimodal transcriptome and epitome sequencing data set (CITE‐Seq) (COMBAT Consortium, 2022), we found that compared to other cell types, monocytes expressed a higher level of both CDKN1A and CD36 (Figure 2g), and those non‐classical monocytes in COVID‐19 patients exhibited higher levels of both markers than in healthy volunteers (Figure 2h).

This study utilized an integrative approach to identify a unique senescent profile at both RNA and protein levels in the blood of SARS‐CoV‐2 patients. Our findings suggest that this senescence signature is predominantly expressed in CDKN1A (p21)‐positive monocytes, which could potentially contribute to the hyper‐inflammatory response observed in severe cases of COVID‐19. Monocytes can be infected by SARS‐CoV‐2, influencing the outcome of the infection through their cytokine production, immune signalling and antigen presentation to T cells (Knoll et al., 2021; Zhou et al., 2020).

If monocytes undergo senescence, it is plausible that their ability to effectively clear the virus and regulate inflammation could be compromised. Additionally, senescent monocytes might exhibit altered cytokine production and immune signalling, leading to dysregulated inflammation during the later stages of the infection. Therefore, senescent monocytes may contribute to the increased severity of COVID‐19. Identification of senescence‐associated markers in circulating monocytes might help to predict severe pathogenesis and potential long‐term pathological sequelae. In addition, targeting senescent monocytes might be a promising approach to mitigating the immune dysregulation and tissue damage associated with infection. However, further research is needed to fully understand the mechanisms underlying induction of monocyte senescence in COVID‐19, the potential role of senescent monocytes in driving disease severity and the potential to develop senotherapeutic strategies targeting monocytes to limit disease severity.

## AUTHOR CONTRIBUTIONS

All authors participated in analysing and interpreting the data. DFP, HRB and MD conceptualized the study and designed the experiments; DFB and HRB curated the biobank; YL performed bioinformatics analysis; YL and LSS performed and analysed the bulk RNA‐Seq experiment; JLK, JME‐N and TK performed and analysed the proteomics experiment; YL, MB and MD wrote the manuscript; all authors revised the manuscript.

## FUNDING INFORMATION

The establishment of Acutelines has been made possible by funds from the University Medical Center Groningen. Work in the laboratory of MD is funded by the Dutch Cancer foundation (KWF) and by the Dutch Research Council (NWO).

## CONFLICT OF INTEREST STATEMENT

The authors declare no conflict.

## Supporting information


Figure S1
Tables S1–S2

Appendix S1

## Data Availability

The data that support the findings of this study are available from the upon reasonable request.
