# Supplementary material for: Circulating monocytes expressing senescence‐associated features are enriched in COVID‐19 patients with severe disease
Source: Aging Cell. 2023 Nov 15;22(12):e14011. doi: 10.1111/acel.14011 (PMC10726854; doi:10.1111/acel.14011)
Supplement: Supplementary file 1 — Figure S1 Tables S1–S2 [file ACEL-22-e14011-s002.zip › acel14011-sup-0005-Legends.docx]

Legends

Figure S1. Violin plot showing the expression levels of cell-type-specific markers from PBMCs.

Table S1. Detailed information of the AcuteLines patients included in the analysis.

Table S2. Differentially expressed genes in white blood cells of patients with different Covid-19 severity.
