# Supplementary figures and images for: Circulating monocytes expressing senescence‐associated features are enriched in COVID‐19 patients with severe disease
Source: Aging Cell. 2023 Nov 15;22(12):e14011. doi: 10.1111/acel.14011 (PMC10726854; doi:10.1111/acel.14011)

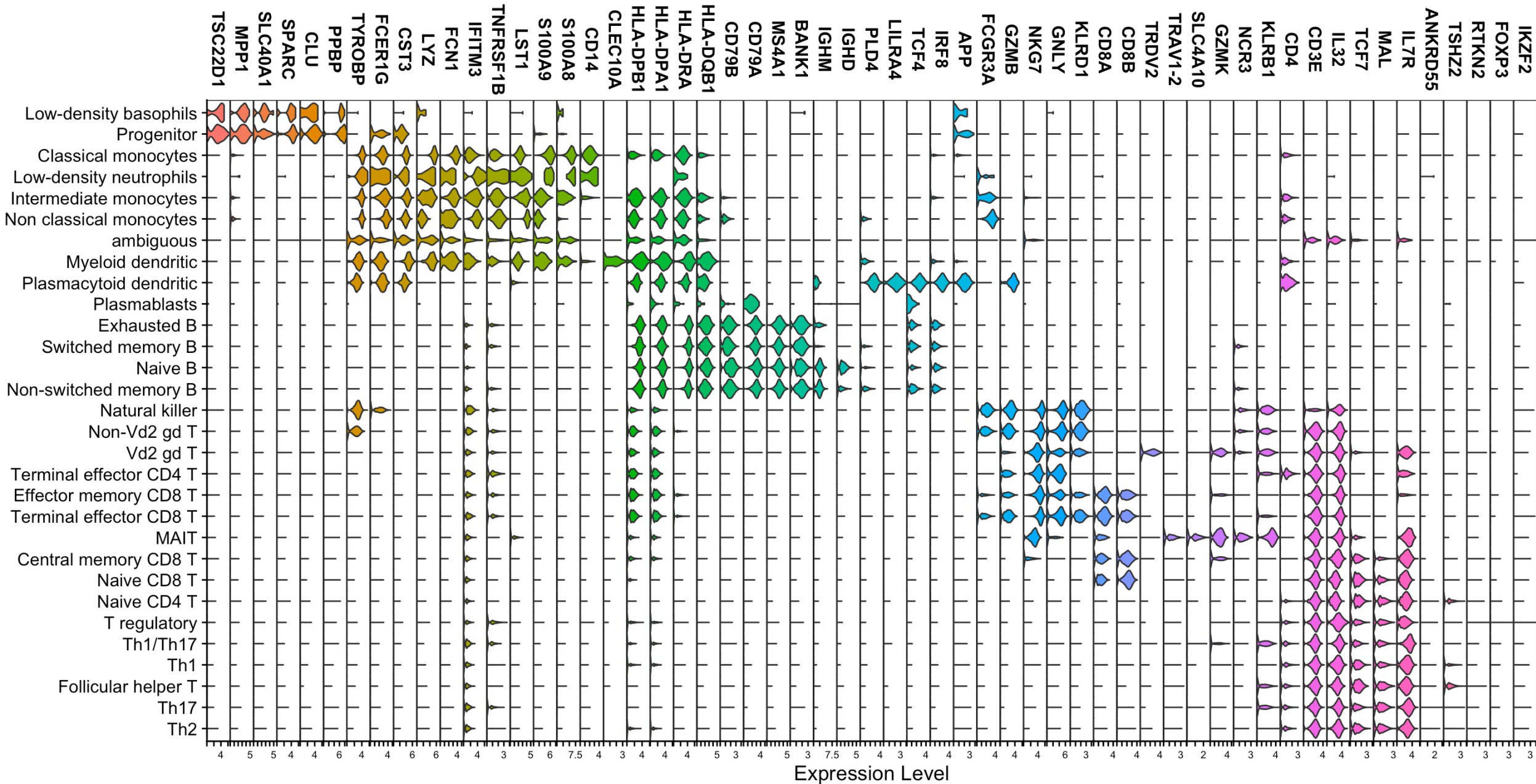

Supplement: Supplementary file 1 — Figure S1 Tables S1–S2 [file ACEL-22-e14011-s002.zip › acel14011-sup-0001-FigureS1.pdf]
