# Supplementary material for: Circulating monocytes expressing senescence‐associated features are enriched in COVID‐19 patients with severe disease
Source: Aging Cell. 2023 Nov 15;22(12):e14011. doi: 10.1111/acel.14011 (PMC10726854; doi:10.1111/acel.14011)
Supplement: Supplementary file 2 — Appendix S1 [file ACEL-22-e14011-s001.docx]

**MATERIALS AND METHODS**

**Patient cohort inclusion**

Data/samples were prospectively obtained by Acutelines: a data-, image and biobank at the Emergency Department (ED) of the University Medical Center Groningen (UMCG)(Ter Avest et al., 2021). In order to allow collection of data and biomaterials when applicable upon first contact, primary screening of patients for eligibility upon arrival in the ED is performed 24/7 by the ED-nurse together with a trained research team. Blood was collected upon triage, before starting treatment, and processed immediately at the ED followed by storage at -80°C. To allow immediate red blood lysis and stabilization of RNA, one blood sample was collected using Qiagen PAXGene tubes. All participants gave (deferred) informed consent (by proxy) on enrolment. Samples were subjected to planar and non-planar immunoassays and bulk RNA-sequencing.

**Plasma SASP measurements**

Measurement of 27 analytes linked to inflammation and Senescence Associated Secretory Phenotype (SASP) was performed at the Facility for Geroscience Analysis (FGA) at Mayo Clinic, an analytical laboratory of the NIH-funded Translational Geroscience Network. MIF, LIF, IGFBP-1, IGFBP3, GM-CSF, GDF-15, TNFRI, Fas/CD95, MCP-1/CCL2, GROα/CXCL1, IP-10/CXCL10, GROβ/CXCL2, IFN-β, IL-1β, IL-2, PDGF-AA, TRAIL/TNFSF10, IL-1α, PAI-1, and TNFRII were measured in EDTA plasma in duplicates using commercially available multiplex magnetic bead immunoassays (R&D Systems) based on Luminex FLEXMAP3D multiplexing platform and IL-6, IL-8, TNFα, IL-7, IGFBP-2, IFNα, and IFNγ were measured using ELLA Simple Plex Platform, with cartridges purchased from Protein Simple/Bio-Techne following manufactures instructions. Analytes with measurements out of the assay range (LIF, GM-CSF, IFN-β, IFNα) were removed for further clustering. The heatmap was clustered using ward.D method, drawn by pheatmap.

**RNA-Seq data sequencing and processing**

To get the gene expression profile of immune cells, we did a whole transcriptome sequencing using the isolated cell fraction. Blood was equilibrated to room temperature for 2 hours before RNA isolation was performed. RNA isolation was performed according to PreAnalitiX PAXgene Blood RNA Kit (ref 762174), followed by RNA quality control (D5000 ScreenTape Assay). Samples were diluted in 100µl RNase free H_2_O to reach a final concentration of 400ng. NEXTflex™ Rapid Directional qRNA-Seq™ Kit was used for the library preparation and the following quality control of libraries was done with Qubit 4 Fluorometer and TapeStation 4200. Libraries were then pooled equal molar to obtain the superpool. Superpool was sequenced on the NextSeq 500 (Illumina).

To obtain good quality and clean reads, we used FastQC (0.11.3)(Wingett & Andrews, 2018) to check the quality of the sequencing reads and Trim Galore (0.6.6) (https://www.bioinformatics.babraham.ac.uk/) to trim the adaptor and poor-quality reads with parameter nextseq 25 and length 36. And then the clean reads were quantified by the Salmon (1.3.0)(Patro et al., 2017) with the parameter gcBias. Transcripts level quantification was summarized to gene level by tximport (1.24.0)(Soneson et al., 2015). We removed samples with extremely low sequencing depth or lying far away from other samples in principle component analysis plot. At next, we used the DESeq2 (1.36.0)(Love et al., 2014) to do the differential expression analysis and ashr (2.2-54)(Stephens, 2017) to shrink the log fold change, with a design to remove the age and sex effect. Benjamini-Hochberg approach was used to correct the multiple testing. Genes with a padj < 0.05 and abs(log2FoldChange) > 0.6 are considered as significantly changed. And then we used the clusterProfile (4.4.4)(Yu et al., 2012) to do the gene set enrichment analysis with compareCluster function.

**Public scRNA-Seq data analysis**

To further investigate the cell types that contribute to the senescence signature in whole blood, we analyzed two public single cell RNA sequencing datasets(Wang et al., 2023; Wang et al., 2022). The first dataset is GSE165080. To better answer our question, we used a subset of cells from moderate and severe COVID-19 patients and healthy controls. At next, we did the basic quality control, including percent of mitochondrial counts less than, number of detected genes larger than 300 and less than 2500, and genes expressed at least in 3 cells, which resulted in 70,158 cells with 34,321 expressed genes. We used scater(McCarthy et al., 2017) to do the log normalization and dimension reduction, used singleR (1.10.0)(Aran et al., 2019) to do the unsupervised cell type clustering with the reference from monos(Monaco et al., 2019). Cell identity were further checked by the marker genes. For example, in monocytes, we checked the expression of LYZ and CD14 gene (for details see Fig. S1). Cell cycle position was estimated with tricycle (1.4.0) (Zheng et al., 2022). SenMayo (Saul et al., 2022) expression score was assessed by AUCell(Aibar et al., 2017) and visualized by ggplot2 (3.4.2) ( [https://ggplot2.tidyverse.org](https://ggplot2.tidyverse.org/)). The second dataset is COMBAT(Wang et al., 2023). For this dataset, we used all the cells from COVID patients and healthy donors, and keep the annotation results by the original paper because of their massive efforts and multi modal data used to annotate. The visualization was done by scanpy(Wolf et al., 2018) python package.

**Availability of the codes and data**

SASP levels and differentially expressed genes are available within the supplementary tables. Raw RNA sequencing data from patients are available upon request. Public datasets used by this project are available from the GEO repository GSE165080 (<https://www.ncbi.nlm.nih.gov/geo/query/acc.cgi?acc=GSE165080>) (Wang et al., 2022) and zenodo (<https://zenodo.org/record/6120249>(Wang et al., 2023). Computational analysis was performed in R (version 4.2.1) and Python (version 3.10.9). Code for analysis and figures are available online at Github (<https://github.com/yaolin101/COVID-19>).

**REFERENCES**

Aibar, S., Gonzalez-Blas, C. B., Moerman, T., Huynh-Thu, V. A., Imrichova, H., Hulselmans, G., Rambow, F., Marine, J. C., Geurts, P., Aerts, J., van den Oord, J., Atak, Z. K., Wouters, J., & Aerts, S. (2017). SCENIC: single-cell regulatory network inference and clustering. *Nat Methods*, *14*(11), 1083-1086. <https://doi.org/10.1038/nmeth.4463>

Aran, D., Looney, A. P., Liu, L., Wu, E., Fong, V., Hsu, A., Chak, S., Naikawadi, R. P., Wolters, P. J., Abate, A. R., Butte, A. J., & Bhattacharya, M. (2019). Reference-based analysis of lung single-cell sequencing reveals a transitional profibrotic macrophage. *Nat Immunol*, *20*(2), 163-172. <https://doi.org/10.1038/s41590-018-0276-y>

Love, M. I., Huber, W., & Anders, S. (2014). Moderated estimation of fold change and dispersion for RNA-seq data with DESeq2. *Genome Biol*, *15*(12), 550. <https://doi.org/10.1186/s13059-014-0550-8>

McCarthy, D. J., Campbell, K. R., Lun, A. T., & Wills, Q. F. (2017). Scater: pre-processing, quality control, normalization and visualization of single-cell RNA-seq data in R. *Bioinformatics*, *33*(8), 1179-1186. <https://doi.org/10.1093/bioinformatics/btw777>

Monaco, G., Lee, B., Xu, W., Mustafah, S., Hwang, Y. Y., Carre, C., Burdin, N., Visan, L., Ceccarelli, M., Poidinger, M., Zippelius, A., Pedro de Magalhaes, J., & Larbi, A. (2019). RNA-Seq Signatures Normalized by mRNA Abundance Allow Absolute Deconvolution of Human Immune Cell Types. *Cell Rep*, *26*(6), 1627-1640 e1627. <https://doi.org/10.1016/j.celrep.2019.01.041>

Patro, R., Duggal, G., Love, M. I., Irizarry, R. A., & Kingsford, C. (2017). Salmon provides fast and bias-aware quantification of transcript expression. *Nat Methods*, *14*(4), 417-419. <https://doi.org/10.1038/nmeth.4197>

Saul, D., Kosinsky, R. L., Atkinson, E. J., Doolittle, M. L., Zhang, X., LeBrasseur, N. K., Pignolo, R. J., Robbins, P. D., Niedernhofer, L. J., Ikeno, Y., Jurk, D., Passos, J. F., Hickson, L. J., Xue, A., Monroe, D. G., Tchkonia, T., Kirkland, J. L., Farr, J. N., & Khosla, S. (2022). A new gene set identifies senescent cells and predicts senescence-associated pathways across tissues. *Nat Commun*, *13*(1), 4827. <https://doi.org/10.1038/s41467-022-32552-1>

Soneson, C., Love, M. I., & Robinson, M. D. (2015). Differential analyses for RNA-seq: transcript-level estimates improve gene-level inferences. *F1000Res*, *4*, 1521. <https://doi.org/10.12688/f1000research.7563.2>

Stephens, M. (2017). False discovery rates: a new deal. *Biostatistics*, *18*(2), 275-294. <https://doi.org/10.1093/biostatistics/kxw041>

Ter Avest, E., van Munster, B. C., van Wijk, R. J., Tent, S., Ter Horst, S., Hu, T. T., van Heijst, L. E., van der Veer, F. S., van Beuningen, F. E., Ter Maaten, J. C., & Bouma, H. R. (2021). Cohort profile of Acutelines: a large data/biobank of acute and emergency medicine. *BMJ Open*, *11*(7), e047349. <https://doi.org/10.1136/bmjopen-2020-047349>

Wang, D., Kumar, V., Burnham, K. L., Mentzer, A. J., Marsden, B. D., & Knight, J. C. (2023). COMBATdb: a database for the COVID-19 Multi-Omics Blood ATlas. *Nucleic Acids Res*, *51*(D1), D896-D905. <https://doi.org/10.1093/nar/gkac1019>

Wang, X., Bai, H., Ma, J., Qin, H., Zeng, Q., Hu, F., Jiang, T., Mao, W., Zhao, Y., Chen, X., Qi, X., Li, M., Xu, J., Hao, J., Wang, Y., Ding, X., Liu, Y., Huang, T., Fang, C., Ge, C., Li, D., Hu, K., Ren, X., Zhang, B., Zhang, B., Shi, B., & Zhang, C. (2022). Identification of Distinct Immune Cell Subsets Associated With Asymptomatic Infection, Disease Severity, and Viral Persistence in COVID-19 Patients. *Front Immunol*, *13*, 812514. <https://doi.org/10.3389/fimmu.2022.812514>

Wingett, S. W., & Andrews, S. (2018). FastQ Screen: A tool for multi-genome mapping and quality control. *F1000Res*, *7*, 1338. <https://doi.org/10.12688/f1000research.15931.2>

Wolf, F. A., Angerer, P., & Theis, F. J. (2018). SCANPY: large-scale single-cell gene expression data analysis. *Genome Biol*, *19*(1), 15. <https://doi.org/10.1186/s13059-017-1382-0>

Yu, G., Wang, L. G., Han, Y., & He, Q. Y. (2012). clusterProfiler: an R package for comparing biological themes among gene clusters. *OMICS*, *16*(5), 284-287. <https://doi.org/10.1089/omi.2011.0118>

Zheng, S. C., Stein-O'Brien, G., Augustin, J. J., Slosberg, J., Carosso, G. A., Winer, B., Shin, G., Bjornsson, H. T., Goff, L. A., & Hansen, K. D. (2022). Universal prediction of cell-cycle position using transfer learning. *Genome Biol*, *23*(1), 41. <https://doi.org/10.1186/s13059-021-02581-y>
